# G2 Dendrimer as a Carrier Can Enhance Immune Responses Against HCV-NS3 Protein in BALB/c Mice

**Published:** 2019

**Authors:** Foozieh Javadi, Pooneh Rahimi, Mohammad Hossien Modarresi, Azam Bolhassani, Mehdi Shafiee Ardestani, Seyed Mehdi Sadat

**Affiliations:** 1. Department of Hepatitis and AIDS, Pasteur Institute of Iran, Tehran, Iran; 2. Depratment of Biology, Science and Research Branch, Islamic Azad University, Tehran, Iran; 3. Department of Medical Genetics, Faculty of Medicine, Tehran University of Medical Sciences, Tehran, Iran; 4. Department of Radiopharmacy, Faculty of Pharmacy, Tehran University of Medical Sciences, Tehran, Iran

**Keywords:** Dendrimers, Hepatitic C virus, Mice, Vaccines

## Abstract

**Background:**

Hepatitis C virus (HCV) infection is a major issue of public health. It seems of paramount importance to find an effective vaccine against HCV infection. The best vaccine candidate should induce robust cellular responses. The aim of the current study was to evaluate immunogenicity effects of novel conjugated dendrimer G2 with the recombinant NS3 antigen as a vaccine candidate for eliciting Th1-oriented cellular responses.

**Methods:**

Female BALB/c mice were immunized with different regimes especially with NS3 conjugated with G2 dendrimer. The humoral responses (Total IgG and IgG iso-typing) and cellular responses (*Ex vivo* IFN-γ and IL-4 ELISpot assays, *in vitro* CTL assay and proliferation) were evaluated and compared in immunized mice.

**Results:**

The results indicated that induced specific total IgG in all mice groups immunized with rNS3 formulated with different adjuvants and IgG2a subclass was the predominant isotype in rNS3-G2 (p≤0.05). For preliminary evaluation of cellular response, *ex vivo* ELISpot assay has shown that the higher frequency of IFN-γ producing cells was in groups immunized with rNS3+M720 and rNS3-G2 (*p*= 0.0012) than control groups. Finally, the rNS3-specific CTLs activity showed the highest percentage of specific lysis (LDH release) of the target cells in rNS3-G2 and rNS3+M720 groups.

**Conclusion:**

In the present study, as our knowledge, this is first time that the immunogenicity of nanodendrimer G2 as a biocompatible adjuvant with the HCV-NS3 antigen was evaluated. The results showed high capability of the regimen to induce strong Th1-orinted cellular response in mice model, indicating the dendrimer G2 as a novel adjuvant candidate for HCV vaccine studies.

## Introduction

Hepatitis C virus (HCV) infection is a major issue of public health. There are more than 180 million people chronically infected with the virus 1,2. HCV is one of the main factors to cause liver cirrhosis and hepato-cellular carcinoma 3. Although the new therapy with direct acting antiviral (DAAs) has an effect on success rate of the treatment, some side effects, cost, availability and low-rate treatment failure still remain the main issues of concern 2,4. To address this concern, finding and developing of prophylactic and/or therapeutic vaccines is necessary 5. HCV is a single positive RNA genome, about 9.6 *kb*, that encodes three structural and six non-structural proteins 6. The NS3 or non-structural protein 3 is a bi-functional protein with serine protease, RNA helicase activity that is involved in HCV replication and translation 7,8. On the other hand, NS3 with the existence of conserved and immuodominat regions, is a main target to the vaccine and antiviral therapy studies 7–12. Several studies have shown the crucial role of cellular immunity in spontaneous virus elimination. So, an effective vaccine against HCV infection should be eliciting robust cellular immune responses 13. In subunit vaccine, recombinant antigens generally induce humoral response and to achieve strong cellular responses, Th1-specific adjuvant formulation is needed 14,15. Current human compatible adjuvants, which lead to poor immune responses and finding new adjuvant with high ability to induce strong and long-term immune responses are necessary 16,17. Recently, nanoparticles were introduced as adjuvants to stimulate both humoral and cellular responses 18–22. Dendrimers are nanoparticle carriers with compact globular structures that have many applications, including biomedical, drug delivery properties and applications in vaccine studies 19,23. In the synthesis of dendrimers, their size and molecular mass can be precisely controlled and the presence of a large number of end branches increases the solubility and reactivity of dendrimers 24,25. In the previous study, new conjugation was synthesized form NS3 protein with dendrimer G2 and in the current study, its immunogenicity in mice model was evaluated.

## Materials and Methods

### Antigens and adjuvants

In the current study, rNS3-HCV and rNS3-G2 conjugates were used as antigens with different regimens. Human compatible adjuvant Montanide ISA 720 (M720, SEPPIC, France) and complete/incomplete Freund adjuvants (C/IFA, Sigma, Aldrich) were used in immunization protocol. Recombinant NS3-HCV (Amino acids 1095-1387) was expressed into *Escherichia coli (E. coli)* BL21-DE3 expression system and high yield of the protein as well as purification under native condition was obtained as described in the previous study 26. Synthesis and conjugation of G2 dendrimer with the recombinant protein (rNS3-G2) was presented as a new formulation with biodegradable properties 24.

### Mice immunization protocols

Female BALB/c mice (Pathogen properties free with 6–8 weeks of age-average, 20 *g* of weight) were purchased from Pasteur Institute of Iran and were used and handled according to the international animal care ethics. Eight groups of seven mice were immunized at weeks 0, 3 and 6 either subcutaneously (*s.c*.) in the tail base with 5 *μg* of rNS3-G2 in 100 *μl* of PBS or with 5 *μg* of rNS3 emulsified in 70% Montanide ISA 720 (M720) 27 or 50% Freund adjuvants. Two weeks after last immunization, the mouse blood samples were collected by retro-orbital bleeding and isolated sera were stored at −70°*C* before experiments.

### Humoral response assay

Antibody (IgG) response of immunized mice was analyzed by ELISA assay. Briefly, purified rNS3 (3 *μg/ml*) was used as a captured molecule to coat 96-well polyvinyl chloride ELISA plates (Nunc, Denmark) for overnight at 4*°C*. After washing and blocking steps, wells were probed with optimum dilution of mice sera (1/1000 for total IgG), incubated for 1 *hr*, washed and further incubated with HRP-conjugated goat anti-mouse IgG (Sigma, Aldrich) as secondary antibody. Finally, by addition of TMB (Tetramethylbenzidine; Roche), color development was measured at 450 *nm*. IgG antibody subclasses were analyzed as mentioned above using goat anti-mouse IgG1, IgG2a, IgG2b antibodies (Sigma, Aldrich) and rabbit anti-goat IgG-HRP conjugate (Sigma, Aldrich). Optimum dilutions of mice sera were determined to achieve measurable ELISA signals against the coated antigen 27.

### Ex vivo ELISpot assay

The frequency of IFN-γ and IL-4 secreting cells were evaluated on the splenocytes of mice in each group using ELISpot kits (MabTech, USA), according to the manufacturer’s instruction. Briefly, first all 96-well plates (Nunc, Denmark) were separately coated with anti-IL-4 or anti-IFN-γ monoclonal capture antibodies for overnight at 4*°C*. Then, the coated plates were washed and blocked with PBS-T and BSA (1% *v/v*), respectively. A 10^5^ splenocytes were seeded per well and co-cultured with 10 *μM* of rNS3-HCV for 40 *hr* at 37°*C* and 5% CO_2_. After washing and removing splenocytes, the plates were incubated with anti-IL-4 and anti-IFN-γ, biotin-conjugate polyclonal antibodies. After washing and incubation with streptavidin-alkaline phosphatase and finally adding BCIP-NBT substrate which led to the appearance of Spot-Forming Cells (SFCs), they were counted under a dissection stereoscope (Leica Microscopy system, Heerbrugg, Switzerland). The wells containing ConA (5 *μg/ml*, Sigma, USA) and irrelevant peptide (132–145 residues from HCV-Core protein; DLMGYIPLVGPLG) served as positive and negative controls, respectively.

### In vitro cytotoxic T-lymphocyte (CTL) assay

The rNS3-specific CTLs were measured using Lactate Dehydrogenase (LDH) assay (Roche, Germany) according to the manufacturer’s instructions. For this purpose, the immunized mice were boosted with the antigen three days prior to isolation of splenocytes as effector cells. The P815 cells (H-2d matched cells) were used as target cells. The effector and target cells were stimulated with rNS3-HCV (10 *μM*) in RPMI-1640 with addition of 10-5 *M* 2-mercaptoethanol (Sigma, USA) and 100 *mM* HEPES for 24 and 72 *hr*, respectively. The cells were co-cultured at the indicated E/T (Effector/Target) cell ratios (100/1, 50/1 and 25/1) for 6 *hr* at 37°*C* and 5% CO_2_. Finally, lytic activity was measured using LDH assay kit at O.D. of 490 *nm*. The defined controls and each experiment were repeated at least three times and finally the amount of specific lysis activity (CTL) was calculated according to the formula by the manufacturer.

### Cell proliferation assays

This assay was carried out using the cell proliferation ELISA, BrdU (5-bromo-2 deoxyuridine) kit (Roche, Germany) according to the manufacturer’s instructions. Briefly, the splenocytes (3×10^5^ cells/well) were co-cultured with 10 *μM* of rNS3-HCV in a 96-well culture plate (Nunc, Denmark) for 60 *hr* at 37°*C* and 5% CO_2_ and further incubated with BrdU solution (10 *μM/well*) for 12 *hr*. After washing and addition of FixDenat, the wells were probed with Anti Brdu –POD antibody for 90 *min*. Finally, proliferation analysis was measured by addition of TMB (Roche, Germany) at O.D. of 490 *nm*. Stimulation Index (SI) was calculated from the absorbance of stimulated/un-stimulated blanks.

### Statistical analysis

All experiments were performed in triplicate and repeated three times. The differences between results were statistically analyzed by Mann–Whitney non-parametric test and one-way ANOVA. p*-*values less than 0.05 (p<0.05) were considered significant.

## Results

### Evaluation of humoral response (Total IgG and subclasses)

**Figure 1.**
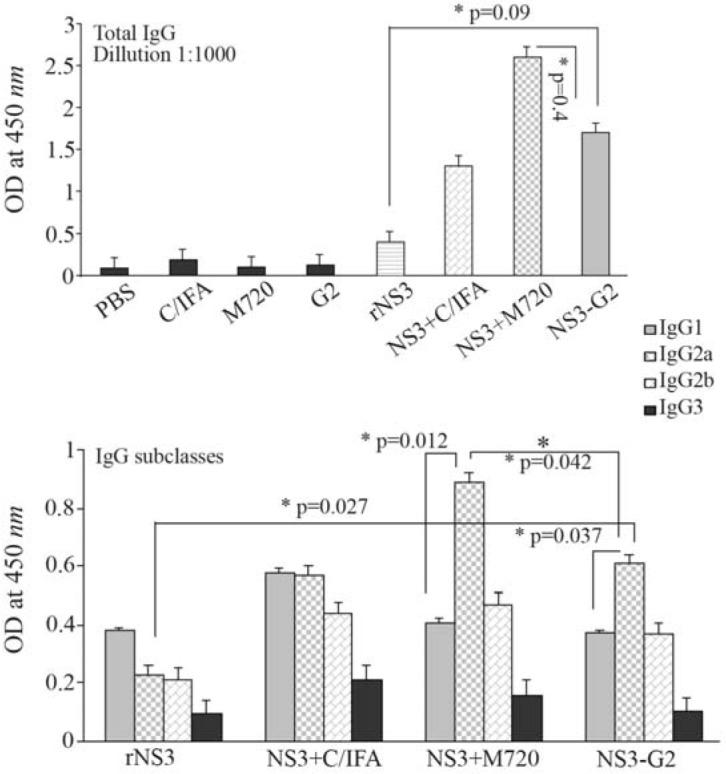
Analysis of humoral responses (Total IgG and IgG subclasses) using ELISA. Each formulation is abbreviated on the horizontal axis of diagrams (See the text for detailed methods). Total IgG was determined at 1:1000 dilution of mice sera, isotype-specific antibodies IgG1, IgG2a, IgG2b and IgG3 were determined at optimum dilution of mice sera that were determined prior to comparisons, by testing serially diluted sera pooled from individual mice of test groups against the coated antigen to achieve measureable ELISA signals. All assays were performed in triplicate and at least for five mice. Error bars are shown as means±SD per groups and * indicates the significant differences.

As shown in figure 1, all mice groups immunized with rNS3 formulated with different adjuvants induced specific total IgG, albeit in different levels, while the level of specific-IgG antibody response significantly increased after the third immunization in comparison with the second injection (Data not shown). Accordingly, mice group vaccinated with rNS3+M720 induced higher levels of total IgG (p=0.00001); the data showed the efficiency of rNS3-G2 regimen in comparison with rNS3 immunization (p=0.00001). Of note, mice groups injected with PBS or adjuvants alone (Negative control groups) did not show any specific reactivity to the HCV-NS3 antigen. The IgG antibody subclasses were analyzed in mice sera at the optimum dilution of total IgG antibody which was O. D=0.5 at 450 *nm*. The data indicated that IgG2a subclass was the predominant isotype (Th1-biased response) in both groups immunized with rNS3+M720 or rNS3-G2 (p≤0.05). In rNS3-G2 group, IgG2a showed significantly higher titers in comparison with mice group vaccinated with rNS3 alone (p=0.0001).

### Evaluation of cellular response (ELISpot assay)

**Figure 2.**
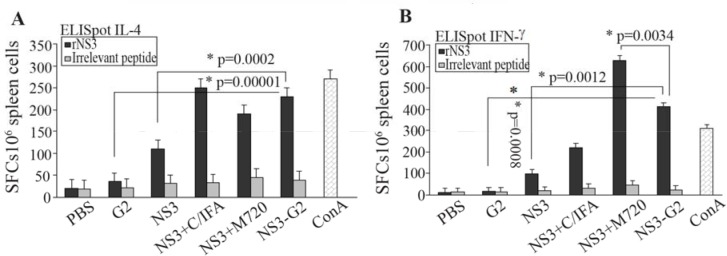
IFN-γ and IL-4 ELISpot assays for immunity responses. The splenocytes were cultured with rNS3 protein (10 *μM*) for 40 *hr*. Spot-forming cells (SFCs) representing the number of IL-4-secreting splenocytes (A) and IFN-γ-secreting splenocytes (B) were quantified under a dissection stereoscope. ConA (5 *μg/ml*) was used as a positive control and irrelevant protein (HIV-NEF) was used as negative control. Error bars are shown as means±SD per groups and *indicates the significant differences. All assays were performed in triplicate and at least for five mice. All abbreviations are listed in methods section.

*Ex vivo ELISpot* assay was performed for preliminary evaluation of cellular response. For this reason, the splenocytes of immunized mice were evaluated to determinate frequency of IFN-γ and IL-4 secreting cells. As shown in figure 2, the higher frequency of IFN-γ producing cells were in mice groups immunized with rNS3+M720 and rNS3-G2 (p= 0.0012) than control groups, respectively. There was no significant difference in the frequency of IFN-γ and IL-4 producing cells in the mice group vaccinated with Freund’s adjuvants. The data confirmed the potency of rNS3-G2 immunization towards the stimulation of a strong cellular immunity. On note, the absence of detectable responses against irrelevant peptide in the experiment indicated the specificity of obtained results.

### Evaluation of rNS3-specific CTL activity

**Figure 3.**
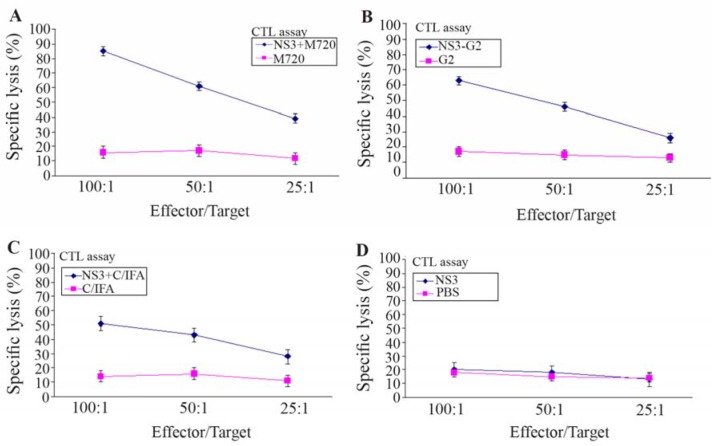
*In vivo* CTL activity assay in immunized mice groups of different formulation. The assay was performed using LDH release ELISA kit. The spleen cells from the mice groups were immunized with, A) NS3 + M720, B) NS3-G2, C) NS3 + C/IFA, D: The rNS3 regimens. All effector and target cells were restimulated by rNS3 antigen. All assays were performed in triplicate and at least for five mice. Error bars are shown as means ± SD per groups and *indicates the significant differences.

The rNS3- specific CTLs activity was evaluated based on LDH release in cell culture supernatant. As shown in figure 3, the CTLs of mice immunized with rNS3-G2 and rNS3+M720 showed the highest percentage of specific lysis of the target cells (P815) at the indicated E/T ratios, respectively. In contrast, there was no detectable specific CTLs response in mice groups that were vaccinated with rNS3 or adjuvants alone. The data is consistent with the results obtained from the *ELISpot* assays in terms of inducing strong cellular immunity.

### Effect of rNS3 antigen on the proliferation of splenocytes in immunized mice

**Figure 4.**
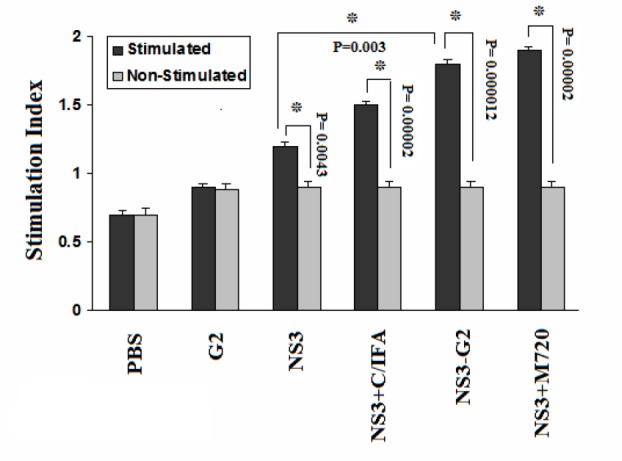
Cell Proliferation Assays. The spleen cells of different immunized mice were restimulated with rNS3 and were measured using BrdU colorimetric ELISA kit. All assays were performed in triplicate and at least for five mice. Error bars are shown as means ± SD per groups and *indicates the significant differences. All abbreviations are listed in materials and methods.

To evaluate lymphocyte proliferation, the splenocytes of immunized mice were stimulated with rNS3 antigen with incorporation of BrdU into the splenocytes detected by ELISA method. The results represented that the mice group immunized with rNS3-G2 had a significantly higher stimulation index (SI) in comparison with control groups (p=0.000012). Also, there was a significant difference between SI of mice group vaccinated with rNS3+M720 and rNS3 alone (p=0.003) (Figure 4).

## Discussion

An effective vaccine against HCV is not currently available and development of either a preventive or at least a therapeutic vaccine is a matter of research focus. Such an effective HCV vaccine should be able to induce potent and broadly cross-reactive CD4+ and CD8+ T-cells, as well as neutralizing antibody (NAbs) responses 28. Aiming for this, the current study sought to evaluate the immunogenicity of rNS3/dendrimer G2 conjugate as a new formulation in BALB/c mouse model. Spontaneous HCV clearance and protection from re-infection have been shown to be associated with potent multi-functional cellular immunity (Th1-biased response) against the viral antigens 13,29. Non-Structural (NS) antigens of HCV have been targeted in several studies seeking for the development of a therapeutic vaccine 30–33. Among NS proteins, NS3 seems to be an ideal target for the purpose of vaccine design 34–36. However, despite these promising results, few studies have shown the immune suppressive role of NS3 when it has been used as a full-length protein 37. As a proof of the argument, more potent immune responses against HCV-NS3 have been achieved by avoiding the NS3 protease activity and selecting the immunogenic regions of the protein 36. Administration of recombinant proteins generally leads to their engulfment by APCs and their delivery to TCD4+ cells *via* the MHC Class-II pathway. Therefore, the initial response to HCV infection is through specific TCD4 + cells, which play a central role in the creation and maintenance of an inherent immune response 38. However, through a mechanism known as cross-priming extracellular antigens, it is possible to induce TCD8+ responses. Cooperation of TCD4+ helper cells promotes the maturation strong response of TCD8+ cells 39. Given the important role of adjuvants for the induction of robust and effective responses, the second-generation G2 dendrimer was used and the NS3-specific induced responses were compared with other commercially available adjuvants. It has also been shown in previous studies that subcutaneous (*s.c*) or intramuscular (*i.m*) injection results in more DCs 40. It has been shown that nanoscale particles of the same size, injected in different locations, cause different stimuli and responses in APCs, which ultimately lead to a difference in immune responses 41. To induce a robust cellular and consequently a humoral NS3-specific response in mouse, the recombinant protein in its native form was purified and formulated with various adjuvants, including the newly introduced dendrimer G2 nanoparticles.

In a previous study, BSA was evaluated as an antigen using PLGA adjuvant and it was shown that particles with a diameter of 1000 *nm* have improved IgG responses in comparison to particles of 200 to 500 *nm*^42^. Kanchan and Panda reported that HBS-Ag antigen in PLA increased the production of IgG levels 43, and in contrast to smaller particles, it has the potential to induce a stronger antibody response, the results of which are presented in the study by Jung *et al* with the formulation of Tetanus Toxoid (TT) with PLGA 44. Wendoref *et al* provided the proper immune response to the ENV of the HIV-1 virus in the form of fusion with MenB Neisseria, in conjunction with PLGA 45. In agreement with a previous report 46, the highest antibody titer was found when the NS3 protein was formulated with M720 adjuvant. Also, the regimen of G2 with NS3 was significantly different (p=0.00001) than the antigens alone group. In another study which used the NS3 antigen with the QuilA adjuvant, a high titer of specific antibodies was obtained 47. The results of this study showed that the induction of antibody production was higher than HBSAg-G2 nanoconjugate 19. In the analysis of IgG subclasses in different groups, the most important subclasses in the antigenic groups with adjuvant M720, and the G2 dendrimer with the NS3 were IgG2a (Th1 response) andIgG1 (Th2 response).

The secretion profile of IFN-γ and IL-4 cytokines was respectively Th1 and Th2 immune responses 27. Our ELISpot assay showed the formation of NS3-specific memory T cells within the spelocytes of the mice immunized with NS3 formulated with various adjuvants. Immunization with NS3 protein alone, due to the presence of TCD4+epitopes, stimulates the helper CD4+cells, which leads to the induction and proliferation of TCD8+cells that ultimately differentiate into functional CTLs and memory cells 48. Secretion of both IFN-γ and IL4 cytokines in animals that were immunized with NS3-G2 regimen was higher than NS3 alone group. In this group, the dominant secretion was IFN-γ, which is another indication for the bias of immune responses to Th1 (Cellular) rather than Th2 (Humoral). Also, nanoparticle G2 adjuvant increased the cellular proliferation significantly compared to the antigenic injection group alone (Figure 4).

The CTL assay showed that using both M720 and G2 dendrimer results in the activation of central memory and functional T cells. However, it seems that the central memory cells were less in frequency in NS3-G2 than NS3+M720 group. The results of our CTL assay in NS3-G2 group indicated a relative increase in cytotoxicity response compared to another study, in which NS3/NS4 proteins were formulated with G2 nanoparticles 49. Induction of cellular immune responses has been well represented in various studies using nanoparticles 43,50. It is shown that particles with dimensions smaller than 100 *nm* produce stranger cellular responses than larger particles 51. Given the small size of the G2 dendrimer, a strong CTL response was expected in NS3-G2 animals compared to the NS3 alone as well as Freund’s adjuvant control group (Figure 3). Induction of Th1-oriented immune responses by the application of 600-200 *nm* particulate adjuvants indicates the capability of this size of nanoparticles for being uptaken by APCs, especially macrophages 43. Our results indicated that immunization of BALB/c mice with NS3-G2 regimen induced considerably strong cellular responses concomitant with borderline humoral reactions. These observations are in consistency with other researches that used cationic polymers with a size of 630-220 for HIV-TAT specific immunization 50.

## Conclusion

Spontaneous HCV clearance is known to be associated with potent multi-functional cellular immunity and TCD8+ responses. Our study for the first time shows that conjugation of nanodendrimer G2 with NS3 HCV antigen is a potent immune stimulator of TCD8+ cells in mouse. This finding warrants further investigations about the contribution of NS3-G2 formulation to immunosurveillance against HCV infection.
